# Multiple sclerosis and obesity: The role of adipokines

**DOI:** 10.3389/fimmu.2022.1038393

**Published:** 2022-11-15

**Authors:** Jorge Correale, Mariano Marrodan

**Affiliations:** ^1^ Departamento de Neurología, Fleni, Buenos Aires, Argentina; ^2^ Instituto de Química y Fisicoquímica Biológicas (IQUIFIB), Universidad de Buenos Aires/Consejo Nacional de Investigaciones Científicas y Técnicas (CONICET), Buenos Aires, Argentina

**Keywords:** adipokines, adipose tissue, multiple sclerosis, neuroinflammation, obesity

## Abstract

Multiple Sclerosis (MS), a chronic inflammatory disease of the central nervous system that leads to demyelination and neurodegeneration has been associated with various environmental and lifestyle factors. Population-based studies have provided evidence showing the prevalence of MS is increasing worldwide. Because a similar trend has been observed for obesity and metabolic syndrome, interest has grown in possible underlying biological mechanisms shared by both conditions. Adipokines, a family of soluble factors produced by adipose tissue that participate in a wide range of biological functions, contribute to a low state of chronic inflammation observed in obesity, and influence immune function, metabolism, and nutritional state. In this review, we aim to describe epidemiological and biological factors common to MS and obesity, as well as provide an update on current knowledge of how different pro- and anti-inflammatory adipokines participate as immune response mediators in MS, as well as in the animal model for MS, namely, experimental autoimmune encephalomyelitis (EAE). Multiple Sclerosis (MS) is a chronic inflammatory disease of the central nervous system (CNS) leading to demyelination, and neurodegeneration. Although its pathogenesis is not yet fully understood, there is considerable evidence to suggest MS arises from complex interactions between individual genetic susceptibility and external environmental factors. In recent decades, population-based studies have provided evidence indicating the prevalence of MS is increasing worldwide, in parallel with the rise in obesity and metabolic syndrome. This synchronous increment in the incidence of both MS and obesity has led to a search for potential biological mechanisms linking both conditions. Notably, a large number of studies have established significant correlation between obesity and higher prevalence, or worse prognosis, of several immune-mediated conditions. Fat tissue has been found to produce a variety of soluble factors named adipokines. These mediators, secreted by both adipocytes as well as diverse immune cells, participate in a wide range of biological functions, further strengthening the concept of a link between immune function, metabolism, and nutritional state. Because obesity causes overproduction of pro-inflammatory adipokines (namely leptin, resistin and visfatin) and reduction of anti-inflammatory adipokines (adiponectin and apelin), adipose tissue dysregulation would appear to contribute to a state of chronic, low-grade inflammation favoring the development of disease. In this review, we present a summary of current knowledge related to the pathological effects of different adipokines, prevalent in obese MS patients.

## Introduction

Multiple sclerosis (MS) is an autoimmune disease of the central nervous system (CNS) characterized by demyelination and neurodegeneration. Its pathogenesis has been associated with the interaction of genetic, environmental and lifestyle factors (1–4). During recent decades, population-based studies have provided evidence indicating an increase in MS incidence worldwide, particularly in women (5–7). Given the short time span during which these changes have occurred, they are difficult to explain based solely on genetic factors, highlighting the potential contribution of the environment and/or lifestyle factors to the phenomenon. Prevalence and incidence of obesity have also significantly increased in the last decades in both sexes and in all age groups, as a result of lifestyle changes (8), to the point of becoming a worldwide health-care crisis (9). In the last three decades, prevalence of obesity has risen on average by 27.5% in adults and 47.1% in children (10), according to different estimates. The observed parallel increase in MS and obesity has led to a search for potential biological mechanisms common to both conditions.

Traditionally, adipose tissue and the immune system have been considered to exist as two entirely separate body systems, with distinct biological functions. However, it is now known that adipose tissue is hormonally active, and secretes cytokines and adipokines. Adipokines are a group of hormone-like molecules produced by white adipose tissue, which exert autocrine and paracrine functions and regulate energy metabolism, inflammation, and immune responses (11). Indeed, the importance of adipose tissue as a secondary immune organ is now becoming increasingly appreciated. Obesity modifies the adipokine profile, shifting it towards a more pro-inflammatory, and less anti-inflammatory state. Indeed, single-cell sequencing analysis of immune cells isolated from human and from mice adipose tissue, show clear differences between samples from lean and obese tissue (12, 13), suggesting the underlying immune state may influence metabolic processes. Obesity can exert several inflammatory effects on the CNS. Saturated fatty acids interacting with the toll-like receptor (TLR)-4, have been shown to induce neuro-inflammatory effects, mediated in part by induction of the nuclear factor kappa-light-chain-enhancer of activated B cell (NF-κB) pathway. Notably, these inflammatory effects can be observed before weight gain in animal models exposed to high-fat diets (14). In mice, adipose tissue can be the source of tumor necrosis factor (TNF)-α, a cytokine which is increased in obesity and alters insulin sensitivity, indicating once again, a link between metabolic and inflammatory regulation (15). Macrophages have been identified as the primary source of TNF-α (16), and several studies have found adipose tissue is host to a variety of different innate and adaptive leukocyte populations (17, 18). After TNF-α was described, an array of other cytokines and chemokines regulating the immune system and metabolism, including interleukin (IL)-6 and monocyte chemoattractant protein-1 (MCP-1), were found to originate from this same source. Evidence is accumulating to suggest that obesity induces a chronic inflammatory state through the activation of TLRs, altering the polarization of innate and adaptive immune cells in different tissues, including the CNS. These changes may contribute to loss of immune self-tolerance in genetically predisposed individuals, triggering pathogenic events such as those associated with MS.

In this review, we will describe epidemiological factors and biological findings present in obesity and in MS, as well as metabolic and immunological effects mediated by different adipokines that could contribute to the development of MS.

## Obesity and multiple sclerosis: Epidemiological links

Different comorbidities such as insulin resistance, type 2 diabetes and immune-mediated diseases have been linked to obesity. Interestingly, obese subjects present more severe forms of autoimmune disease, and in general show poorer therapeutic response (19–21).

Elevated body mass index (BMI) and obesity play a major role in MS development. Studies have shown that BMIs = 30 kg/m^2^ in adolescence, not only increase the risk of developing MS (**Table 1**), but have been associated with greater levels of disability, as well as increased neuroinflammation and gray matter atrophy (22, 23). Although early studies presented limitations in their design (retrospective, self-reported weight/height), findings were later confirmed in a prospective longitudinal study, which found a 1.6-1.9-fold increase in risk of developing MS in young obese individuals between the ages of 7 and 13 years. This association was significantly stronger in girls than in boys (24). An increased risk of pediatric MS has also been observed in extremely obese girls (BMI > 35 kg/m2; 25) presenting an isolated demyelinating event, including optic neuritis, brainstem syndrome, and transverse myelitis. In yet another longitudinal study, each 1 kg/m^2^ increase in BMI, was independently associated with a reduction in both normalized gray matter volume, as well as brain parenchyma, although a significant clinical association between BMI elevation and a greater degree of disability was not clearly established (23).

**Table 1:** Epidemiological data published on increased risk of Multiple Sclerosis in obese patients.

Author, year of publication	Study design	Country	Number of MS patients/total population or controls	F:M	Period of life evaluated	Results
Munger KL et al, 2009	Retrospective cohort	USA	593/238371	1	C, A, EA	Increased risk of developing MS in obese adolescents
Hedström AK et al, 2012	Case-Control	Sweden	1571/3371	3:1	A, EA	BMI above 27 linked to increased risk of developing MS
Munger KL et al, 2013	Prospective cohort	Denmark	774/302043	2:1	C	Higher risk of developing MS in girls
Langer-Gould A et al, 2013	Case-Control	USA	75/913097	2:1	C	Girls with obesity present increased risk of MS
Gianfrancesco MA et al, 2014	Case-Control	USA	1235/697	4:1	C, A, EA	Higher BMI correlates with MS risk, particularly in women
Wesnes K et al, 2014	Case-Control	Norway, Italy	1160/3050	2:1	C, EA	Obesity in C and A increased risk of developing MS
Hedström AK et al, 2014	Case-Control	Sweden, USA	2447/2626	2:1	A, EA	Interactions between BMI and HLA genotype increase MS risk
Kavak SK et at, 2015	Retrospective cohort	USA	184*	1**	A, EA	Patients with increased BMI in A and EA develop MS earlier
Hedström AK et al, 2015	Case-Control	Sweden	2055/4518	NA	C, A	BMI during adolescence, rather than C, is critical in determining MS risk.
Chitnis T et al, 2016	Case-Control	USA	254/420	2:1	C, A	Adolescents with higher BMI had increased risk of MS. Earlier age at sexual maturity associated with obesity significantly increased risk of developing early MS
Huppke B et al, 2019	Restrospective cohort	Germany	524/15271	1,5:1	C, A	Obesity increased risk of developing MS in both genders. Obese patients, had statistically significant more relapses on first-line treatment***
Høglund RAAa et al, 2021	Prospective cohort	Norway	1409/648734	2:1	A, EA	High BMI increased MS risk
Marrodan M et al, 2021	Case-Control	Argentina	309/322	3:1	A, EA	Excess weight and obesity increased risk of developing MS.

A, adolescence; BMI, Body mass index; C, childhood; EA, early adulthood; F:M, female:male ratio; HLA, human leukocyte antigen; MS, multiple sclerosis *Only patients with MS were evaluated. **Exclusively women with MS were included. *** First-line treatment: Interferon or glatiramer acetate

There is also evidence to suggest obesity interacts with individual genetic factors to increase susceptibility to MS, and significant interaction has been observed between HLA-DRB1*15 allele presence and obesity, in relation to risk of MS. In young adults with BMIs under 27 kg/m^2^ who carried the allele DRB1*15 but lacked the protective allele A*02, a 5.1-fold increased risk of developing MS was observed, whereas in individuals with this same genotype but a BMI ≥27 kg/m^2^ this increase skyrocketed to 16.2 times greater risk (25)

To date, information on the association between obesity and MS progression remains scarce, and preliminary studies on BMI as a predictor of disability have shown contradictory results (22, 26–29) possibly because of differences in the disability assessment scales used. It is important to consider whether in patients with MS, obesity reflects a simple overlapping of conditions, or exerts direct effects favoring disease development.

## Biological links between MS and obesity

The stroma of lean adipose tissue is made up of regulatory T cells (Treg cells), invariant natural killer cells (iNKT cells), M2 macrophages, natural killer cells (NK cells), innate lymphoid cells type 2 (ILC2), and eosinophils, all contributing to creating an anti-inflammatory environment. Obesity modifies this environment towards a more pro-inflammatory one, represented by a significant increase in M1 macrophages, as well as recruitment, and proliferation of neutrophils, CD8^+^ T cells, T helper 1 cells (Th1 cells). At the same time, a decrease in iNKT cells, ILC2 cells, and Treg cells, Th2 immunosuppressive mediators (e.g., IL-4, IL-10, TGF-β) occurs, together with impaired expression of peroxisome proliferator-activated gamma (PPAR-γ) which plays an essential role in maintaining adipose tissue homeostasis. Overall, this imbalance induced a low-grade chronic inflammatory environment, which determines a local and systemic dysregulation of the immune system, creating the perfect environment for the development of autoimmune disorders. It also alters different metabolic pathways, in particular the one linked to insulin resistance (17, 18, 30, 31).

**Figure 1:**
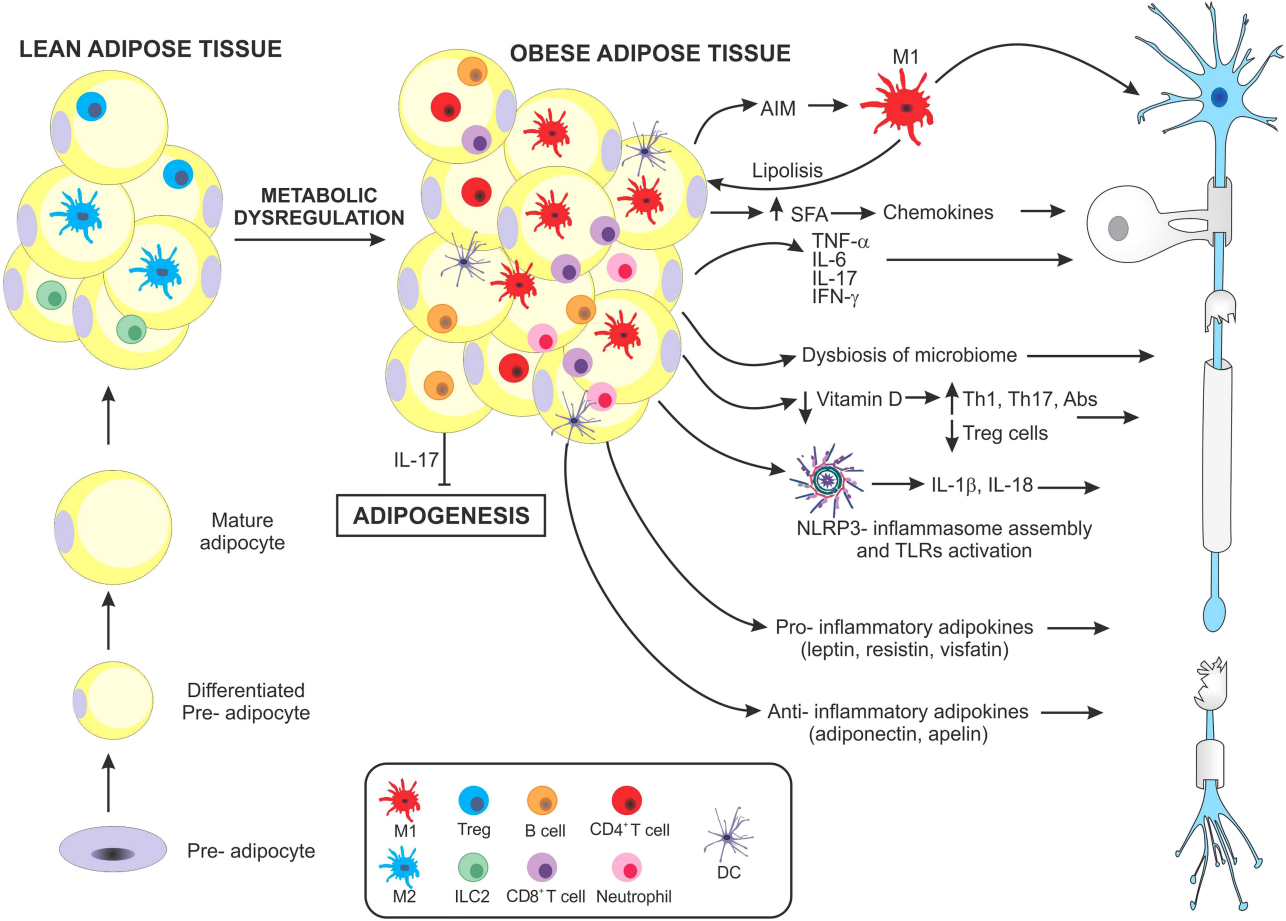
Schematic representation of main mechanisms suggested to promote multiple sclerosis in obesity. As obesity develops, hypertrophic adipocytes and changes in immune cell populations contribute to the development of a chronic inflammatory microenvironment. Both adipocytes and immune cells massively infiltrating adipose tissue secrete high levels of different molecules that favor a pro-inflammatory state. Macrophages in obese adipose tissue produce the apoptosis inhibitor of macrophage factor, which promotes macrophage survival, against various apoptosis-inducing stimuli from adipocytes. The apoptosis inhibitor of macrophages induces lipolysis, increasing saturated fatty acid levels, which in turn perpetuate pro-inflammatory M1-macrophage infiltration. Saturated fatty acids can activate the assembly of the NLRP3-inflammasome, which secretes IL-1β and IL-18, both involved in pro-inflammatory pathogenesis. In addition, both adipose tissue and infiltrating immune cells secrete several pro-inflammatory cytokines including, TNF-α, IL-6, IFN-γ, and IL-17 all involved in inducing MS. Paradoxically, IL-17 has also been shown to inhibit adipogenesis. Another factor influenced by obesity is the contribution of nutrients, especially a decrease in Vitamin D and changes occurring in the gut microbiome. These in turn may induce profound modulations in the balance of Th17/Treg cells. Finally, the adipose tissue of obese individuals produces particular types of adipokines which have been linked to different autoimmune diseases, including MS, some with pro-inflammatory, and others with anti-inflammatory effects.

Several theories have been postulated to explain underlying immune mechanisms promoting obesity-associated autoimmune diseases, including MS (**Figure 1**).

First, the presence of a macrophage-derived molecule, the apoptosis inhibitor of macrophage (AIM) which improves macrophage survival, also induces lipolysis of adipose tissue, preventing the progression of obesity. However, when lipolysis is excessive, saturated fatty acid release may cause chemokine production in both adipocytes and resident macrophages, *via* Toll-like receptor (TLR)-4, resulting in additional M1 macrophage recruitment. These mechanisms favor a paracrine/autocrine induced inflammatory response (32–34). AIM, a secretory protein, is also found in peripheral blood. As obesity progresses, it binds to the Fc fraction of IgM, increasing its stability and circulating levels. This obesity-induced rise in IgM has been associated with increased production of IgG autoantibodies (35, 36). Second, serum and adipose tissue levels of the pro-inflammatory cytokines IL-6 and TNF-α are high in obese mice and overweight individuals, in correlation with increased insulin resistance (37). During the transition from a lean to an obese phenotype, infiltrating anti-inflammatory CD4+ T cells decrease, and pro-inflammatory T cells (e.g., Th1 and Th17) predominate. In murine studies, diet-induced obesity can promote Th17-biased immunity, partly dependent on IL-6 (20). Interestingly, gene-expression heat maps showed lean mice expressed the Th17-lineage defining transcription factor *Rorc*, but not the effector cytokines *Il17a, Il17f, or Il22* or the *Il23r*. In contrast, obese mice expressed *Rorc*, the effector cytokines, and the *Il23r*, determining a more mature population of Th17 cells (19). Similarly, in humans, an immune profile biased by Th17 cells has been observed in obese individuals (38), particularly by isoforms IL-17A and IL-17F, which are central mediators of inflammation and may contribute to the development of MS (39). In addition, IL-17 can also affect metabolic homeostasis by inhibiting adipogenesis (40), favoring an increase in circulating free fatty acids, thus worsening insulin resistance and CNS inflammation.

Third, increased inflammation affects the blood-brain barrier (BBB), causing a decrease in tight junction proteins and the process of transcytosis (41–43). As a result, there is increased leukocyte extravasation, with upregulation of vascular cell adhesion molecule 1 (VCAM-1), intercellular adhesion molecule 1 (ICAM-1), P-selectin, and E-selectin (44, 45). In addition, both TNF-α and IL-1β induce the expression of the chemokines CXCL1 and CCL2, which further increase immune cell recruitment (46).

Fourth, the microbiomes of obese and lean people differ in striking ways. It is conceivable that dietary components modify the composition and functional status of the host microbiome (47). The microbiome of obese patients can profoundly modulate extraintestinal immune responses through different mechanisms. For example, lipopolysaccharides (LPS) from the outer membrane of Gram-negative bacteria can cross the intestinal mucosa and consequently produce systemic inflammation (48). The intestine of people with obesity has also shown increased permeability compared to lean people (47), and metabolites from the gut microbiota can enter the bloodstream and directly modulate the host’s immune system (49). Consequently, the Treg/Th17 cell ratio may be altered, inducing functional phenotypes in T helper cells that affect the development of neuroinflammation (50).

Fifth, there has been consistent association in several studies between increased BMI and decreased levels of 25 hydroxy (25OH) vitamin D. Possible mechanisms for low 25OH Vitamin D in obese individuals include: restricted dietary intake, less exposure to sunlight, accelerated metabolic clearance, or more widespread distribution of 25OH vitamin D (51). Several authors have shown that 25OH Vitamin D regulates immune mechanisms that may be important in MS, namely inhibition of pathogenic Th1 and Th17 cell differentiation, increased sensitivity to apoptotic mechanisms of CD4^+^ effector T cells, and increased numbers, as well as heightened suppressive function of Treg and Tr1 cells (52).

Sixth, obesity can induce inflammasome assembly and activation of NOD receptor-like protein 3 (NLRP3). This process primarily involves the binding of ceramides, fatty acids, oxidized low-density lipoproteins, and cholesterol crystals produced in obese patients through binding to TLR 2/4, as well as an increase in the levels of inflammasome effectors: pro-IL-1β, and pro-IL-18. Following activation, oligomerization of the inflammasome occurs, leading to cleavage of pro-caspase-1 to active caspase-1, which in turn can cleave both pro-IL-1β and pro-IL-18 into bioactive forms, which then enter the circulation or local extracellular environment (53).

Finally, numerous studies have documented that overweight and obesity are associated with impaired secretion of adipokines, which can exert autocrine and paracrine functions and regulate several metabolic and immunological processes (11).

## Adipokines

The adipose tissue is both an energy storage site, and a hormone-secreting organ (54). Different proteomic studies have shown that the molecules it produces are mainly peptides, grouped under the name adipokines, that can exert both pro-inflammatory and anti-inflammatory effects (55–57). Their secretion profile is modified by adipocyte hypertrophy, making them not only important modulators of adipose tissue per se, but also of numerous physiological functions in other target organs, including the brain, liver, muscle, vasculature, heart, pancreas, as well as the immune system (58). Dysregulation of adipokine secretion may therefore be involved in MS pathophysiology, increasing risk of disease development in obese individuals, as well as dampening treatment response (59–61). **Table 2** summarizes the main effects of different adipokines on innate and adaptive immunity, as well as results observed in studies in the experimental autoimmune encephalomyelitis (EAE) model and in MS patients.

**Table 2:** Overview of major immune effects of adipokines, and their influence on EAE and MS.

Adipokine	Innate immune system effects	Adaptive immune system effects	Effects on EAE	Effects on MS
Pro-inflammatory				
Leptin	-Enhances proliferation and phagocytic activity of macrophages, as well as secretion of IL-1β, IL-6, and TNF-α. -Induces expression of IL-1β, and TNF-α on microglia cells.	-Increases thymocyte maturation. -Stimulates proliferation of Th1 cells and production of IFN-≤, and IL-2. -Suppresses production of IL-4 and IL-10, and inhibits proliferation of Treg cells	-Leptin deficient mice are resistant to both passive and active induction of EAE. - Leptin receptor blockade prevents migration of immune cells into the CNS, and disease progression -Administration of leptin worsens EAE, while LEPR ameliorates disease. -Acute starvation attenuates clinical symptoms and delays disease onset.	-In RRMS patient serum and CSF, levels are increased in correlation with increased IFN-≤ levels, and reduction of Treg cells. -Serum leptin levels and EDSS correlate positively in SPMS and PPMS -Although some controversies persist, the general trend in the data points toward increased levels of leptin in MS, associated with increased disease burden - The age of onset and sex conditions differences in leptin levels
Resistin	-Increases production of IL-1β, IL-6, TNF-α, IL-12, and MCP-1 by macrophages	-Increases production of IL-1β, IL-6, IL-12 and TNF-α.	N/A	Increased serum levels found in RRMS patients, correlates positively with pro-inflammatory cytokines and EDSS, and negatively with the expression of FoxP3 mRNA of T cells
Visfatin	-Induces synthesis of IL-1β, IL-6, and TNF-α Chemotactic factor by upregulation of MCP-1, CXCL2 and CXCL8. -Induces synthesis of IL-1β, Il-6, iNOS, NO, and ROS in microglial cells. -Induces CD54, CD40 and CD80co-stimulatory molecules	-Increases maturation of T cells, and production of IL-1β, IL-6, IL-12, TNF-α.	-Inhibition after symptom onset significantly reduced the disability and demyelination of the spinal cord. - *In vitro* studies have demonstrated suppression of T cell proliferation and depletion of T cells,	Increased serum levels in RRMS compared to SPMS and PPMS, positive correlation with TNF-α and IL-1β, and negative correlation with the expression of FoxP3 mRNA of T cell
Chemerin	-Chemoattraction of dendritic cells and macrophages -During resolution, polarizes macrophages towards an anti-inflammatory phenotype.	Induces expression of CMKLR1 in lymphocytes	-Up-regulates CMKLR1 in lymphocytes infiltrating the spinal cord. -CMKLR1 KO mice develop less severe disease. -Involved in lymphocytes and dendritic cells migration - CMKLR1 antagonist, inhibits the infiltration of the CNS by inflammatory cells in EAE animals, reduces the myelin damage, and delays the onset of the disease.	- Probable increase in plasma levels observed in obese RRMS patients. - CMKLR1+ leukocytes, and dendritic cells are identified in the leptomeninges and in the perivascular cuffs of active and chronic MS lesions
Adipocyte fatty-acid binding protein 4	Contributes to differentiation of monocytes into macrophages.	N/A	-Knockout mice exhibit reduced clinical symptoms of EAE and impaired pro-inflammatory cytokine production by dendritic cells.	-High plasma levels in SPMS and pediatric MS patients. - Higher levels have been observed preferentially in females, -High plasma levels are associated with increased disability independent of BMI
Anti-inflammatory				
Adiponectin	-Inhibits maturation, proliferation and phagocytic activity of macrophages, as well as synthesis of TNF-α and IFN-≤ -Increases production of IL-10, IL-1RA, and TGF-β	-Increase number and proliferation of Treg cells -Enhances apoptosis and inhibits proliferation of Ag-specific T cells -Decreases B cell lymphopoiesis -Polarizes Th1 and Th17 cells	-Adiponectin deficient mice present increased CNS inflammation, demyelination and axonal injury -*In vivo* anti-inflammatory effects -Calorie restrictions attenuates EAE which is associated to increased levels of adiponectin	-Serum levels are decreased in RRMS patients, particularly in male. In other studies increase has been linked to early onset MS and remission -Higher levels found during remission compared to relapse phase -Higher levels found in CSF during remission - High and middle molecular weight isoforms of adiponectin were associated with a greater risk of disease progression and severity
Apelin	-Down-regulates expression of IL-6, TNF-α, MCP-1, MIP-1, ROS, and phagocytic activity in macrophages. -N9 microglia cell lines: decreases iNOS and IL-6, and up-regulates IL-10 and arginase.	Anti-inflammatory effects on macrophages Pro-inflammatory effects on microglia cells	-Apelin/APJ receptor complex expression in oligodendrocytes is correlated with age-associated changes in remyelination efficiency - Promotes the differentiation of neural stem cells	- Lower levels in RRMS compared to controls. -Statistically significant negative correlation with disability scores and the number of relapses -Decreases in a very early stages of MS in females, with positive correlation to level of disability and number of relapses

APJ, Apelin receptor; BMI, Body mass index; CRP: CXCL, Chemokine (C-X-C motif) ligand; CMKLR1, Chemerin Chemokine-Like Receptor 1; CNS, central nervous system; CSF, cerebrospinal fluid; EAE, Experimental autoimmune encephalomyelitis; EDSS, Expanded Disability Status Scale; FoxP3, Forkhead box 3; IFN, Interferon; IL, Interleukin; IL-1RA, IL-1 receptor antagonist; iNOS, inducible nitric oxide synthase; LEPR, Leptin receptor; MCP-1, Monocyte chemoattractant protein-1; MIP-1, macrophage inflammatory protein 1; MyD88, Myeloid differentiation primary response 88; N/A, data not available; NLRP3, NOD-; LRR- and pyrin domain-containing protein 3; NO, nitric oxide; PPMS, primary progressive multiple sclerosis; ROS, reactive oxygen species; RRMS, Relapsing-remitting multiple sclerosis; SPMS, secondary progressive multiple sclerosis; TGF-β, transforming growth factor β; Th, T helper cell; TNF, Tumor necrosis factor; Treg cells, regulatory T cells. TLR4, Toll like receptor-4; TXNIP, thioredoxin-interacting protein.

## Pro-inflammatory adipokines

### Leptin

Leptin is the principal regulator of body weight and is produced by adipose tissue when body energy needs are met. It acts on specific hypothalamic nuclei, inducing secretion of an anorexigenic neuropeptide, pro-opiomelanocortin, and of the suppressing orexigenic neuropeptide Y (62, 63). This makes it the most important regulator of body weight, producing satiety and inducing energy expenditure, its levels correlating with body adipose mass and BMI (64). Encoded by the LEP gene (homologous to the ob gene in mice) leptin is mainly produced by adipocytes and secreted into circulation. Its activity is exerted through a receptor, a member of the Class I cytokine family (LEPR or Ob-R) (65). Six isoforms del receptor LEPR with different physiological roles have been identified (66). After binding to its receptor, leptin activates a tyrosine kinase, Janus kinase 2 (JAK2), subsequently leading to phosphorylation of an extracellular signal-regulated kinase (ERK), and the signal transducer and activator of transcription (STAT)3 and STAT5. Later activation of cytokine signaling-3 (SOCS3) results in negative feedback which inhibits the LEPR (67). Notably, LEPR is expressed in CD4^+^ T cells, CD8^+^ T cells, Treg cells and NK cells (68–70). Aside from playing an important role in energy homeostasis, leptin is a potent modulator of the immune response, playing a significant role in regulating leukocyte extravasation in the CNS. Leptin receptor blockade prevents migration of immune cells into the CNS, attenuating EAE progression (71). Moreover, starvation produces a significant decrease in leptin levels, associated in turn with infection, as a result of a dysfunctional immune response, and reversed by exogenous administration of leptin (72). Leptin acts directly on macrophages, enhancing their proliferation and phagocytic activity, as well as the secretion of pro-inflammatory cytokines such as TNF-α, IL-1 and IL-6 (73, 74). Likewise, leptin stimulates proliferation of Th1 cells as well as production of IFN-γ and IL-2, suppressing production of IL-4 and IL-10. Additionally, in rat microglia, leptin induces the expression of IL1-β and TNF-α and enhances LPS effects (75). Leptin also inhibits proliferation of Treg cells and induces hypo-responsiveness (76), effects dependent on increased activity of the mammalian target of rapamycin (mTOR) signaling pathway. Transient inhibition of mTOR by rapamycin prior to TCR stimulation, makes Treg cells proliferate in the absence of IL-2, and recover their inhibitory capacity (69, 77).

Overall, these findings suggest leptin, under certain underlying metabolic conditions coinciding with the loss of immune self-tolerance, can modulate the immune response towards a more pro-inflammatory profile. Leptin-deficient (ob/ob) mice show increased susceptibility to infections, and resistance to induction of both active and passive EAE, associated in turn with progressive decline in autoreactive CD4^+^ T cell survival and reduced IFN-γ and IL-17 production (68). These effects were combined with the down-regulation of protein Bcl-2 survival and cell cycle arrest, determined by reduced degradation of p27 kip1 and impaired signaling of energy-sensing AKT-mTOR pathways (78). Administration of leptin to EAE-susceptible mice (no leptin deficient) worsened the clinical course, while anti-leptin-receptor antibodies ameliorated it (79). Also, prior to the onset of neurological symptoms following EAE induction, animals typically lost body weight, and show a marked increase in serum leptin levels. Immunohistochemical analysis has revealed parallel, *in situ* production of leptin in inflammatory infiltrates and in neurons, occurring only during acute/active phases of EAE (80). On the other hand, starvation delayed disease onset and attenuated clinical symptoms (80). In line with these results, studies in RRMS patients demonstrated increased levels of leptin in serum and cerebrospinal fluid (CSF), and transcriptional analysis of MS brain lesions showed increased leptin expression at sites of inflammation (81). These findings coincided with increased CSF IFN-γ concentrations, and reduced number of circulating Treg cells (82). Serum leptin levels and EDSS results have shown positive correlation in SPMS and PPMS patients (83, 84). Interestingly, in young boys with MS presenting increased leptin levels, longer periods between relapses were observed. Whereas in girls, a positive link between higher leptin levels and increased disability scores was recorded, suggesting varying, gender-specific leptin effects (61). In patients experiencing RRMS exacerbations, increased leptin receptor expression has been reported on CD8+ T cells and monocytes, compared to those in patients in remission or healthy controls (85). In summary, although several studies have found increased leptin levels in MS patients, signaling the hormone could somehow influence burden of disease, it is also important to note they are not strictly comparable, as many did not include important variables affecting the outcome (BMI, gender, or use of DMTs).

### Resistin

Resistin is produced during adipocyte differentiation and was initially described as an adipokine, linking obesity to insulin resistance (86). Resistin increases the expression of cytokines and adhesion molecules in murine vascular endothelial cells, and has been associated with atherogenesis (87). Notably, resistin is mainly produced by adipocytes in rodents, whereas in humans it is produced for the most part by macrophages, and peripheral blood monuclear cells (PBMCs; 91). Human and rodent resistin share only 59% amino-acid identity (88), but their function is similar, despite differences in production source. Resistin increases production of TNF-α, IL-1β, and IL-6, in PBMCs, and macrophages. Conversely, exposure of PBMCs to TNF-α, but not IL-6 or IL-1β, induces expression of resistin (89, 90), further enhancing its own activity through positive feed-back. It also promotes expression of cell adhesion molecules, including VCAM-1, ICAM-1, and MCP-1, and the chemokine (C-C motif) ligand 2 (CCL2), contributing to chemotaxis and recruitment of leukocytes to inflammation sites (87, 91–93). As mentioned above, resistin induces its own production in PBMCs through positive feedback, which is why different studies have highlighted its pro-inflammatory role, by binding to TLR4 and activating NF-κB in human macrophages. In mice hypothalamic cells, its action is exerted *via* c-Jun N-terminal kinase (JNK) p38 and mitogen-activated protein kinase (MAPK) pathways (94). Given its role in insulin resistance and inflammation, resistin is likely to explain, at least in part, the relationship between the inflammatory processes observed in obesity and metabolic diseases.

Increased serum levels of resistin, leptin, and visfatin, as well as decreased expression of forkhead box P3 (FoxP3) mRNA in T cells,have been observed in MS patients, correlating with circulating levels of TNF-α, and IL-1β (95), as well as with disability progression (96), indicating resistin could be an important driver of chronic inflammation. These results support the view that resistin plays a role in the pathogenesis of MS, although additional studies are required to confirm the findings.

### Visfatin

Visfatin is highly expressed by both leukocytes and adipocytes. It is the rate-limiting step in a salvage pathway of nicotinamide adenine dinucleotide (NAD; 101), and in this manner regulates intracellular metabolism. Visfatin binds to the insulin receptor, triggering insulin-mimetic activities. However, it also binds to TLR4, inducing a pro-inflammatory response (97). Visfatin synthesis is promoted by IL-6 and TNF-α (98, 99), while the PPAR-γ agonist rosiglitazone inhibits synthesis by adipocytes (98).

Visfatin exerts pro-inflammatory effects through different mechanisms. In both humans and mice, it induces synthesis of TNF-α, IL1β and IL-6 (100) and acts as a chemotactic factor for monocytes and lymphocytes, by upregulating the chemokines CCL2, CXCL2 and CXCL8 expression in endothelial cells, as well as expression of adhesion molecules like ICAM-1, and VCAM-1 (101). Visfatin also mediates LPS-induced synthesis of IL-6, IL-1β, inducible nitric oxide synthase (iNOS), nitric oxide and reactive oxygen species (ROS) in microglial cells (102). Moreover, it promotes activation of T cells by inducing the expression of co-stimulatory molecules CD54, CD40 and CD80 on monocytes (100). Finally, in mouse endothelial cells, visfatin downregulates expression of tight junction-associated proteins such as zonula occludens 1 and 2, cadherin, and occludin, and therefore increases BBB permeability (103).

In EAE, the inhibition of visfatin after symptom onset significantly reduces disability and demyelination of the spinal cord. Furthermore, *in vitro* studies have demonstrated suppression of T cell proliferation and depletion of T cells, probably through induced inhibition of NAD+ and subsequent ATP depletion (104). In MS patients, levels of visfatin are increased, particularly in RRMS cases, correlating positively with TNF-α and IL-1β levels and negatively with mRNA FoxP3 expression in T cells. Circulating levels of visfatin in RRMS patients are significantly higher compared to those observed in SPMS and PPMS patients (95).

### Chemerin

Chemerin, an adipokine synthesized as a precursor in the liver and in adipocytes, regulates adipocyte differentiation, and is associated with obesity and metabolic syndrome (105). Different chemerin isoforms exert contrasting effects on immune cells, making it difficult to establish their exact role in autoimmune responses. It has been identified as a chemoattractant for plasmacytoid dendritic cells and macrophages, activated *via* TLR9 and alarmin high-mobility group box 1 (HMGB1) factor, to produce type I interferons, which in turn induce pro-inflammatory polarization of adipose tissue (106). In addition, chemerin can also bind to the chemokine receptor CCRL2, which when deficient, promotes macrophage infiltration of adipose tissue and accelerates insulin resistance (107). In the CNS, it is expressed on endothelial cells in the meninges as well as in white matter MS lesions, whereas its receptor, the chemerin chemokine-like receptor 1 (CMKLR1), a G protein-coupled receptor, is expressed mainly on infiltrating lymphocytes, dendritic cells and macrophages. These observations suggest chemerin is involved in processes leading to migration of peripheral cells into the CNS, contributing to the inflammatory process. Indeed, CMKLR1+ leukocytes, and dendritic cells can be found in the leptomeninges and perivascular cuffs of active and chronic MS lesions (108). Chemerin is up-regulated in the spinal cord of EAE animals, and CMKLR1 KO mice develop less severe EAE, failing to induce EAE adoptive transfer (109, 110). Similarly, 2-(alpha-Naphthoyl) ethyltrimethylammonium iodide (α-NETA), a CMKLR1 antagonist, inhibits infiltration of the CNS by inflammatory cells in EAE animals, reducing myelin damage, and delaying onset of disease. It also alters leukocyte distribution in peripheral lymphoid organs (111). Collectively, these findings suggest chemerin is a pro-inflammatory adipokine, possibly contributing to inflammation in obese MS subjects. Increased levels of chemerin have been associated with obesity and excess weight in patients with MS compared to non-obese MS patients and healthy controls, particularly females (112, 113). Conversely, more recent studies have shown that although obese MS patients also present insulin resistance, chemerin levels are not influenced by BMI, nor are chemerin levels related to disease progression, or cognitive dysfunction (114). It has been postulated that chemerin competes with resolvin (an anti-inflammatory molecule synthesized from ω-3 polyunsaturated fatty acids) for binding to the CMKLR1 receptor, a factor promoting resolution of the anti-inflammatory process (115). In the initial stages of inflammation, chemerin stimulates different immune cells on site. During the resolution phase, resolvin activates the CMKLR1 receptor, and macrophages increase production of IL-10, generating an anti-inflammatory profile (116). This mechanism may represent a chemerin/CMKLR1/resolvin control loop, through which chemerin regulates its pro- and anti-inflammatory effects.

### Adipocyte-fatty acid binding protein 4

Adipocyte-fatty acid binding protein 4 (A-FABP; also known as FABP-4 and adipocyte protein 2, aP2) is produced by adipocytes, monocytes and macrophages, and its expression is enhanced by TLR2 stimulation (117). FBPA4 regulates lipolysis and FBPA4 deficiency diminishes pro-inflammatory cytokines, *via* attenuation of IKK-β/NF-κB pathway (118). Conversely, recombinant FBPA4 promotes secretion of pro-inflammatory cytokines in adipocytes *via* the p38/NF-κB pathway (119), supporting its pro-inflammatory role in obesity. Given that FBPA4 is strongly regulated by lipolysis, it could function as a specific lipid sensor in adipocytes, transporting certain plasma lipids to specific organs, including the CNS.

FBPA4 knockout mice exhibit reduced clinical symptoms of EAE and impaired pro-inflammatory cytokine production by dendritic cells (120, 121). Likewise, in MS, FBPA4 has been associated with increased disability (122). Indeed, high plasma levels have been found in SPMS, suggesting a possible pathogenic role for this protein (119). Increased levels of FBPA4 have also been reported in pediatric-onset MS (61, 119). Interestingly, a positive correlation between FBPA4 and leptin in pediatric RRMS patients was reported, suggesting that in the initial stages of the disease, both adipokines play a role in early inflammation as well as in later progression (119). Higher FBPA4 levels have been observed in females, as in the case of leptin (61), and differential expression of microRNAs of FABP4 has been considered a prognostic biomarker of RRMS (123).

## Anti-inflammatory adipokines

### Adiponectin

Adiponectin is the most abundant circulating adipokine. It regulates glucose and lipid metabolism through fatty acid oxidation and inhibition of gluconeogenesis *via* activation of the AMP-activated kinase (AMPK) pathway (124). The latter in turn activates PPAR-γ and PPAR-α signaling. Adiponectin is produced as a monomer, circulating as three different oligomers of varying molecular weight (125). Adiponectin mediates its actions through 3 receptors: AdipoR1, which is located predominantly in skeletal muscle; AdipoR2, expressed mainly in the liver; and T-cadherin, which mediates its actions in the cardiovascular system (126). AdipoR1 and AdipoR2 are also found on monocytes, human B, and NK cells, but only on a small percentage of T cells (127). Adiponectin binding to AdipoR1 promotes activation of the AMPK pathway, while AdipoR2 activates the PPARα pathway (128). Contrary to leptin, adiponectin is decreased in obese individuals, and increases with weight loss (129). While leptin induces pro-inflammatory activity, adiponectin exhibits anti-inflammatory effects on the cells of the immune system. At the endothelial level, adiponectin significantly reduces expression of: TNF-α-induced ICAM-1, VCAM-1, and endothelial-leukocyte adhesion molecule (E-selectin), limiting leukocyte rolling and adhesion (130, 131). In contrast to leptin, adiponectin inhibits maturation, proliferation and phagocytic activity of macrophages, as well as synthesis of IFN-γ and TNF-α after stimulation with LPS (132–134). Its anti-inflammatory effect is mediated by increased production of IL-10 (particularly by Treg cells), as well as of the IL-1 receptor antagonist, and of TGF-β release by monocytes, dendritic cells and macrophages. It also increases Treg cell numbers (134, 135). Notably, overexpression of adiponectin is associated with induction of Th2 and Treg responses *via* PPRA−γ pathway activation, consequently amplifying IL-4 and IL-10 production (136). In addition, anti-inflammatory effects of adiponectin also include enhancement of apoptosis and inhibition of proliferation of antigen-specific T-cells, and inhibition of IL-2-induced NK cell cytotoxic activity (137, 138). However, adiponectin also appears to exert pro-inflammatory effects, activating dendritic cells, and leading to Th1 and Th17 polarization (120). This dual activity (anti- and pro-inflammatory) might depend on specific levels of different circulating isoforms.

In EAE, adiponectin-deficient mice develop more severe disease. T cells from these animals produce greater amounts of TNF-α, IFN-γ, IL-17, and IL-6, and fewer Treg cells, suggesting adiponectin exerts anti-inflammatory effects *in vivo*. Administration of exogenous adiponectin increases Treg cell numbers, ameliorating disease severity (139), whereas calorie-restricted diets decrease EAE severity and are associated with higher plasma adiponectin levels and lower concentration of leptin (140).

Some studies found lower serum levels of adiponectin in males with RRMS compared to healthy controls, with no difference found in females, suggesting adiponectin levels are gender-dependent (61, 101). Conversely, in other cohorts, a predominant decrease in adiponectin was found only in females (141). Some of these discrepancies can probably be explained by differences in age of the populations studied. In studies conducted in twins, higher levels of adiponectin were detected in the CSF of symptomatic MS siblings during remission, compared to the asymptomatic twin. CSF levels did not correlate with serum levels, suggesting either intrathecal synthesis or increased transport across the BBB following enhanced systemic production (142). Unfortunately, these results could not be reproduced by other authors. Larger, more extensive studies will be needed to clarify the real significance of adiponectin in MS.

Some authors observed the presence of high and middle molecular weight isoforms of adiponectin at the time of MS diagnosis, which were associated with a greater risk of disease progression and severity (143, 144) subsequent to IL-6 secretion by monocytes (145). Oligomerization and secretion of adiponectin during inflammation can be modulated by TNF-α (146). Therefore, depending on the isoform, adiponectin may exert pro- or anti-inflammatory effects. All these findings contribute to keeping the discussion open on the significance of adiponectin as a biomarker in MS.

### Apelin

Apelin plays an important role in macrophage function and development in different tissues. In rodents, it down-regulates IL-6, TNF-α and MCP-1 expression in macrophages, as well as macrophage inflammatory protein (MIP)1, ROS formation, and phagocytic activity (147–149). In N9 microglia cell lines, it decreases LPS-induced iNOS and IL-6 production, while up-regulating production of IL-10 and Arginase 1 (150). Overall, apelin is considered an anti-inflammatory adipokine. Even though apelin has not yet been studied in EAE, it has been reported to suppress neuroinflammation in experimental ischemic stroke, by suppressing microglia recruitment and reducing ROS production (151, 152). Neuroprotective effects of apelin on brain ischemia appear to be mediated by the nuclear erythroid 2-related factor (Nrf-2), which is also the target of dimethyl fumarate, a treatment shown to reduce MS relapse rates and disability progression (152). In MS patients, plasma levels of apelin were significantly lower compared to healthy controls, showing a statistically significant negative correlation with disability scores and the number of relapses (153). In addition, apelin and its APJ receptor are widely expressed in the central nervous system, especially in neurons and oligodendrocytes. Notably, in recent studies, apelin/APJ complex expression in oligodendrocytes correlated with age-associated changes in remyelination efficiency through the translocation of myelin regulatory factors (154). Apelin promotes the differentiation of neural stem cells (155) and can therefore represent not only an anti-inflammatory factor but also contribute to repair processes observed during the course of MS.

## Conclusions and future perspectives

Substantial evidence indicates obesity is a risk factor for various autoimmune diseases, including MS. Furthermore, adipose tissue has recently been recognized as an active endocrine organ capable of inducing chronic inflammation through adipokines. Adipokines are not only secreted by adipocytes. Other populations of both innate and adaptive immune cells produce them. They show a broad spectrum of effects, further strengthening the link between immune function, metabolism, and nutritional state. Obesity also induces systemic polarization of immune cells, mediated partially by adipokines. The discovery of pathways linking metabolism and autoimmunity increases our understanding of the relationship between MS and certain lifestyle factors. Leptin and adiponectin have been the most extensively studied adipokines. Information on others such as resistin, chemerin, visfatin, FABP4 and apelin is limited, making it difficult to draw firm conclusions, and comparisons between studies are frequently confounded by factors such as BMI, age, sex, and treatments, all of which significantly impact adipokine levels.

Different adipokines could represent biomarkers of neuroinflammation or neurodegeneration. Although most studies have not considered correlation with other better-validated markers, longitudinal adipokine monitoring ​​could provide more precise information regarding their potential in this sense.

The exact pathophysiology by which adipokines may contribute to MS onset or progression is not fully understood, but the better characterization of how these hormones exert their effects may make them, or their receptors, future therapeutic targets of interest.

Existing studies suggest adipokines exert downstream effects *via* the activation of different signaling pathways, some of which impact adipose tissue and the immune system. These include classic pathways such as AMP-activated protein kinase (AMPK)/mTOR/NF-κ B, p38 MAPK, and Wnt proteins. Our group, for example, has demonstrated that metformin (an agonist of the AMPK pathway), and pioglitazone (a PPAR-γ agonist), two compounds commonly used to treat metabolic syndrome, show beneficial anti-inflammatory effects in MS (156). Dietary interventions can also have an impact on the course of the disease. In animal models, high-fat diets leading to metabolic syndrome increase levels of pro-inflammatory adipokines, which in turn increase severity of EAE, while caloric restriction reduces symptoms and inflammatory infiltration (140).

In time, adipokines or their receptors could become potential targets to treat MS and other inflammatory diseases, although application will be challenging given the extensive network of signaling pathways they work through, and their multiple and often opposing effects at varying sites throughout the human body.

## Author contributions

JC: Conceptualization, Resources, Writing - original draft, Supervision. MM: Investigation, Writing - original draft. All authors contributed to the article and approved the submitted version.

## Acknowledgments

The authors are grateful to Ms. Adriana Zufriategui for the design and drawing of the figure

## Conflict of interest

The authors declare that the research was conducted in the absence of any commercial or financial relationships that could be construed as a potential conflict of interest.

## Publisher’s note

All claims expressed in this article are solely those of the authors and do not necessarily represent those of their affiliated organizations, or those of the publisher, the editors and the reviewers. Any product that may be evaluated in this article, or claim that may be made by its manufacturer, is not guaranteed or endorsed by the publisher.
